# Silencing of the Wheat Protein Phosphatase 2A Catalytic Subunit TaPP2Ac Enhances Host Resistance to the Necrotrophic Pathogen *Rhizoctonia cerealis*

**DOI:** 10.3389/fpls.2018.01437

**Published:** 2018-10-31

**Authors:** Xiuliang Zhu, Yuanyuan Wang, Zhenqi Su, Liangjie Lv, Zengyan Zhang

**Affiliations:** ^1^The National Key Facility for Crop Gene Resources and Genetic Improvement, Institute of Crop Science, Chinese Academy of Agricultural Sciences, Beijing, China; ^2^Institute of Chemistry, Chinese Academy of Sciences, Beijing, China; ^3^Institute for Cereal and Oil Crops, Hebei Academy of Agriculture and Forestry Sciences, Shijiazhuang, China

**Keywords:** protein phosphatase 2A, *Rhizoctonia cerealis*, *Triticum aestivum*, virus-induced gene silencing, reactive oxygen species

## Abstract

Eukaryotic type 2A protein phosphatases (protein phosphatase 2A, PP2A) consist of a scaffold subunit A, a regulatory subunit B, and a catalytic subunit C. Little is known about the roles of PP2Ac proteins that are involved in plant responses to necrotrophic fungal pathogens. Sharp eyespot, caused by the necrotrophic fungus *Rhizoctonia cerealis*, is a destructive disease of wheat (*Triticum aestivum*), an important staple food crop. Here, we isolated *TaPP2Ac-4D* from wheat, which encodes a catalytic subunit of the heterotrimeric PP2A, and characterized its properties and role in plant defense response to *R. cerealis*. Based on the sequence alignment of *TaPP2Ac-4D* with the draft sequences of wheat chromosomes from the International Wheat Genome Sequencing Consortium (IWGSC), it was found that *TaPP2Ac-4D* gene is located on the long arm of the wheat chromosome 4D and has two homologs assigned on wheat chromosomes 4A and 4B. Sequence and phylogenetic tree analyses revealed that the TaPP2Ac protein is a typical member of the PP2Ac family and belongs to the subfamily II. *TaPP2Ac-4B* and *TaPP2Ac-4D* displayed higher transcriptional levels in the *R. cerealis*-susceptible wheat cultivar Wenmai 6 than those seen in the resistant wheat line CI12633. The transcriptional levels of *TaPP2Ac-4B* and *TaPP2Ac-4D* were significantly elevated in wheat *R. cerealis* after infection and upon H_2_O_2_ treatment. Virus-induced gene silencing results revealed that the transcriptional knockdown of *TaPP2Ac-4D* and *TaPP2Ac-4B* significantly increased wheat resistance to *R. cerealis* infection. Meanwhile, the transcriptional levels of certain pathogenesis-related (PR) and reactive oxygen species (ROS)-scavenging enzyme encoding genes were increased in *TaPP2Ac-*silenced wheat plants. These results suggest that TaPP2Ac-4B and TaPP2Ac-4D negatively regulate defense response to *R. cerealis* infection possibly through modulation of the expression of certain *PR* and ROS-scavenging enzyme genes in wheat. This study reveals a novel function of the plant PP2Ac genes in plant immune responses.

## Introduction

In eukaryotic cells, type 2A protein phosphatases (protein phosphatase 2A, PP2A) are a group of serine/threonine (Ser/Thr) phosphatases. They are regulated by reversible protein phosphorylation and are functionally involved in various biochemical processes (Yu et al., 2005). The PP2A proteins are heterotrimeric holoenzyme complexes and are composed of a scaffold or hook subunit A, a regulatory subunit B, and a conserved catalytic subunit C (Durian et al., 2016). According to the structural characteristics, the regulatory subunits B are further subdivided into B, B′, and B″ (Farkas et al., 2007), all of which are conserved among eukaryotes (Booker and DeLong, 2017). The catalytic subunit C shares high similarity with the catalytic subunits of other Ser/Thr protein phosphatases, such as PP1, PP4, and PP6 (Moorhead et al., 2009). In *Arabidopsis* and tomato (*Lycopersicon esculentum*), there are at least five genes encoding PP2A catalytic subunits (PP2Ac) (Kerk et al., 2002; He et al., 2004). Based on the identity among protein sequences, these PP2Ac proteins are grouped into two subfamilies; *AtPP2Ac1*, *AtPP2Ac2*, *AtPP2Ac5*, *LePP2Ac1*, and *LePP2Ac2* belong to subfamily I, while *AtPP2A-3*, *AtPP2A-4*, *LePP2Ac3*, *LePP2Ac4*, and *LePP2Ac5* belong to subfamily II (He et al., 2004).

Recent studies indicate that PP2Ac proteins are involved in abiotic stress signaling in several crop species. For instance, in potato (*Solanum tuberosum*) and rice (*Oryza sativa*), the expression of *StPP2Ac1*, *StPP2Ac2a*, *StPP2Ac2b*, *StPP2Ac3*, *OsPP2A-1*, and *OsPP2A-5* was significantly upregulated by salinity stress (Yu et al., 2003, 2005; País et al., 2009). In tomato, LePP2Ac1, LePP2Ac2, and LePP2Ac3 played an important role in host response to cold stress (País et al., 2009). Type 2A protein phosphatases have also been implicated in plant-biotic interactions. In *Nicotiana benthamiana*, knockdown of homologous genes of *LePP2Ac1* and *LePP2Ac2* led to reduced protein phosphatase activity, upregulation of *PR* genes, growth inhibition of the bacterial pathogen *Pseudomonas syringae*, and increased plant hypersensitive responses to *P. syringae* and *Cladosporium fulvum* (He et al., 2004). In *Arabidopsis*, a holoenzyme PP2A, comprised of the scaffold subunit A1, the regulatory B subunits B’η/ζ, and the catalytic subunit C4, modulated the phosphorylation status of the receptor-like kinase BRI1-ASSOCIATED KINASE1 (BAK1) and controlled the activation of pattern-recognition receptor (PRR) complexes (Segonzac et al., 2014). Accordingly, knock-out of *pp2a-c4* and *pp2a-a1* increased the resistance to *P. syringae* pv. *tomato* DC3000 (Segonzac et al., 2014). In plants, PP2A proteins were also involved in jasmonic acid (JA), auxin, abscisic acid (ABA), and brassinosteroid signal pathways (Garbers et al., 1996; Pernas et al., 2007; País et al., 2009; Tang et al., 2011; Ballesteros et al., 2013; Waadt et al., 2015). For instance, StPP2Ac2 mediated JA signaling and response to wounding stress (País et al., 2009). Recent studies indicated that PP2A subunits were responsive to reactive oxygen species (ROS) signaling and regulated pro-oxidant and antioxidant enzymes at transcriptional and post-transcriptional levels (Strodtkoetter et al., 2009; Trotta et al., 2011; Li et al., 2014; Konert et al., 2015a; Rahikainen et al., 2016). A previous study showed that in a *catalase* (*cat2*) *pp2a-b*′γ double mutant, ROS-induced resistance responses were repressed through the pathways that require PP2a-b′γ activity (Li et al., 2014). However, little is known about the involvement of PP2A in defense responses of an important staple crop, wheat (*Triticum aestivum*), against fungal pathogens.

Common wheat provides dietary carbohydrates for more than one-third of the population in the world (Zou et al., 2018). Production of wheat is critical for global food security, but its grain yield and quality are negatively affected by various pathogens. Among these, the necrotrophic fungus *Rhizoctonia cerealis*, which causes wheat sharp eyespot, is one of the most destructive pathogens of wheat around the world (Chen et al., 2008; Hamada et al., 2011; Lemañczyk and Kwaśna, 2013). After *R. cerealis* infection, the transport tissues in sheaths and stems of wheat can be destroyed, resulting in blocked transportation of substances that are required for nutrition, lodging, and even dead spikes (Chen et al., 2008). Utilization of resistant wheat cultivars is the most economic and effective strategy to control wheat sharp eyespot. Therefore, molecular breeding is important to uncover the mechanism of wheat defense against *R. cerealis* and to identify new resistance regulators from the wheat genome. Previous studies in Zhang’s laboratory have identified several genes that are involved in wheat defense response against *R. cerealis*, such as *TaPIE1* (Zhu et al., 2014), *TaAGC1* (Zhu et al., 2015), *TaCAD12* (Rong et al., 2016), and *TaRCR1* (Zhu et al., 2017). However, no study about the role of PP2A in wheat defense responses to pathogens has been reported yet.

In this study, we cloned a wheat catalytic subunit of the heterotrimeric PP2A gene named as *TaPP2Ac-4D* and investigated its functional role in defense response against *R. cerealis*. *TaPP2Ac-4D* is located on wheat chromosome 4DL and has two homologs that are located on wheat chromosomes 4A and 4B, respectively. The transcriptional levels of *TaPP2Ac-4B* and *TaPP2Ac-4D* were upregulated after *R. cerealis* infection and exogenous H_2_O_2_ treatment. Most importantly, silencing the enhanced resistance of *TaPP2Ac-4B* and *TaPP2Ac-4D* to *R. cerealis* infection upregulated the transcriptional levels of *PR2* and ROS-scavenging genes (*TaCAT1* and *TaAPX2*) in wheat. This study reveals a novel function of plant *PP2Ac* genes in the regulation of resistance responses to fungal pathogens.

## Materials and Methods

### Plant and Fungal Materials and Treatments

Three wheat lines/cultivars, including sharp eyespot-resistant CI12633 and susceptible Wenmai 6 and Yangmai 16, were used in this research. The pathogenic *R. cerealis* strain, WK207, which possesses high-virulence and is prevalent in North China, was provided by Prof. Jinfeng Yu of Shandong Agricultural University, China.

All plants of wheat cultivars Yangmai 16, CI12633, and Wenmai 6 were grown in field plots or in a greenhouse under 14-h-light (22°C)/10-h-dark (12°C) conditions (Zhu et al., 2017). For transcription pattern analysis of *TaPP2Ac* response to *R. cerealis*, wheat plants at the tillering stages of CI12633 and Wenmai 6 were inoculated with *R. cerealis* strain WK207 using the toothpick inoculation method (see below). Samples were collected from stems of wheat plants at 1, 2, 4, 7, 14, and 21 days post inoculation (dpi) with *R. cerealis*. Also, wheat plants treated by toothpick without *R. cerealis* mycelia at each time point were used as mock-inoculation. For expression analysis of *TaPP2Ac* response to H_2_O_2_ treatment, seedlings at the three-leaf stage of Yangmai 16 plants were sprayed with 0.1% Tween-20 solution containing 10 mM H_2_O_2_. The plants sprayed only with water containing 0.1% Tween-20 served as a control. Samples (leaves from five plants were quickly cut and pooled as one sample) were collected at 0, 0.5, 1, 3, 6, and 12 h post treatment (hpt) and were stored at -80°C.

### DNA and RNA Extractions

Genomic DNA for each sample was extracted from 0.1 g of wheat leaves using the CTAB method described by Saghai-Maroof et al. (1984).

To analyze the tissue specific expression of *TaPP2Ac*, samples (0.1 g for each) were harvested from sheaths, stems, leaves, and spikes and were used for RNA extraction. For expression profile analysis of *TaPP2Ac*, ROS-related and PR genes, total RNA was extracted from 0.1 g of wheat stems using TRIzol reagent (Invitrogen) and was purified with RNase-free DNase I (Promega) according to the manufacturer’s instructions.

### Cloning and Sequence Analysis of *TaPP2Ac*

To clone *TaPP2Ac* from wheat, based on the sequence of EF101900.1 (a wheat PP2Ac gene), a pair of primers (TaPP2Ac-FL-F and TaPP2Ac-FL-R, Supplementary Table S1) were designed, synthetized, and used to amplify the full-length sequences from the cDNAs of wheat CI12633, Yangmai 16, and Wenmai 6 stems that were infected with *R. cerealis* isolate WK207 for 7 days. Full-length sequences of *TaPP2Ac-4A*, *TaPP2Ac-4B*, and *TaPP2Ac-4D*, and other *PP2Ac* genes were identified from Ensembl Plants^1^. The theoretical iso-electric point (pI) and molecular weight (MW) of the deduced TaPP2Ac protein sequence was analyzed with DNAMAN. The online smart software package^2^ was used to predict conserved motifs. Multiple protein sequence alignment was carried out using the DNAMAN software. A phylogenetic tree was constructed by using the MEGA6 software.

### cDNA Synthesis, RT-PCR, and qRT-PCR

The purified RNA samples (4 μg each) were separately reverse-transcribed to cDNA using a PrimeScript^TM^ RT Rreagent Kit with gDNA Eraser (TaKaRa, Japan) according to the manufacturer’s protocol. The transcription patterns of BSMV coat protein (*cp*) gene, *TaPP2Ac*, ROS-related and *PR* genes (the primers in Supplementary Table S1) were analyzed by RT-PCR and qRT-PCR, respectively. An ABI 7500 RT-PCR system (Applied Biosystems, United States) was used to operate the qRT-PCR. The total volume of reaction mixture for PCR was 20 μl, which contained 10 μl 2× SYBR Premix Ex Taq, 0.4 μl 50× ROX Reference Dye II, 0.4 μM of each primer, and 5 μl 50-fold diluted cDNA template. The following PCR conditions were used: 95°C for 30 s, followed by 40 cycles of 95°C for 5 s, 58°C for 15 s, and 72°C for 34 s. The relative expression levels of the target genes were calculated using the 2^-ΔΔCT^ method (Livak and Schmittgen, 2001). The wheat *Actin* gene, *TaActin* (GenBank accession number BE425627), was used as an internal reference. The specificity of qRT-PCR products was determined according to the melting curve. The amplification efficiency of qRT-PCR primers were calculated. Three biological replications were performed. Student *t*-test and Dunnett’s test were used to determine the significant differences between controls and treatments.

### *TaPP2Ac* Gene Silencing in Two Wheat Lines by VIGS

Barley stripe mosaic virus (BSMV)-based virus-induced gene silencing (VIGS) technique is a fast and effective reverse genetic tool for researching gene functions (Scofield et al., 2005; Zhou et al., 2007; Zhu et al., 2014, 2015, 2017). To generate the BSMV:TaPP2Ac recombinant construct, a 256 bp sequence of *TaPP2Ac-4D* (from 732 to 987 nucleotides in *TaPP2Ac-4D* cDNA sequence) was amplified using the primers TaPP2Ac-γ-F and TaPP2Ac-γ-R (Supplementary Table S1) and was sub-cloned in an antisense orientation into the *Nhe* I restriction site of the RNAγ of BSMV. The Si-Fi software (Nowara et al., 2010^3^) was used for off-target prediction for the VIGS construct. Capped *in vitro* transcripts were prepared from linearized plasmids containing the tripartite BSMV genome using the RiboMAX^TM^ Large Scale RNA Production System-T7 kit (Promega) and the Ribo m^7^G Cap Analog (Promega) according to the manufacturer’s instructions. The resistant wheat line CI12633 and susceptible wheat cultivar Yangmai 16 were, respectively, used as host to perform the VIGS. At the three-leaf stage, the third fully expanded leaves of wheat plants were inoculated with BSMV:TaPP2Ac or BSMV:GFP (as a control) by gently sliding pinched fingers from the leaf base to the tip for a total of six times. After incubation for 48 h in a humid environment, seedlings were transferred to a greenhouse under 14-h-light (22°C)/10-h-dark (12°C) conditions. After 10 dpi with BSMV, the fourth leaves were collected to monitor BSMV infection based on the transcription of the BSMV *cp* gene. After 3 days of inoculation with *R. cerealis*, the stems were collected to evaluate the transcriptional levels of *TaPP2Ac*, ROS-related, and *PR* genes. The experiment was repeated three times. For one repeat, at least 20 plants were infected by BSMV:GFP and BSMV:TaPP2Ac, respectively. After the experiment, all the BSMV-infected wheat plants were burned down, and the seeds were stored in the refrigerator (-20°C).

### Inoculation With *R. cerealis* and Scoring Response of Wheat Plants

According to Zhu et al. (2017), two methods including wheat kernels inoculation method and toothpick inoculation method were used to inoculate wheat plants. For wheat kernels inoculation method, at the tillering growth stage, CI12633, Yangmai 16, and Wenmai 6 wheat plants were inoculated on each base-stem with 8∼10 sterile wheat kernels harboring the *R. cerealis* isolate WK207 mycelia. For the toothpick inoculation method, at 20 days after BSMV infection, each stem of the BSMV-infected plants was inoculated with one sterile toothpick harboring the *R. cerealis* WK207 mycelia. The *R. cerealis*-infected plants were sprayed with water twice a day during the first week and then once a day until final disease recording. The infection types (ITs) of wheat plants for each line/cultivar were evaluated at 21 dpi with *R. cerealis*, respectively, based on the disease lesion squares on the base stems. The ITs were categorized from 0 to 5 (i.e., “IT:0” = no lesion, “IT:1” = the lesion appeared on the sheaths rather than stems, “IT:2” = the width of the lesion <25% of the infected stem perimeter, “IT:3” = the width of the lesion >25 and <50% of the infected stem perimeter, “IT: 4” = the width of the disease lesion more than 50% of the infected stem perimeter, “IT: 5” = white spike or dead plant).

### Statistical Analysis

To determine the significant differences between controls and treatments or between time-course points, Microsoft Excel was used to calculate mean values and standard deviation and to perform Student *t*-test. The SPSS 19.0 statistical software was used to perform Dunnett’s test.

## Results

### Identification and Sequence Characterization of *TaPP2Ac* in Common Wheat

**FIGURE 1:**
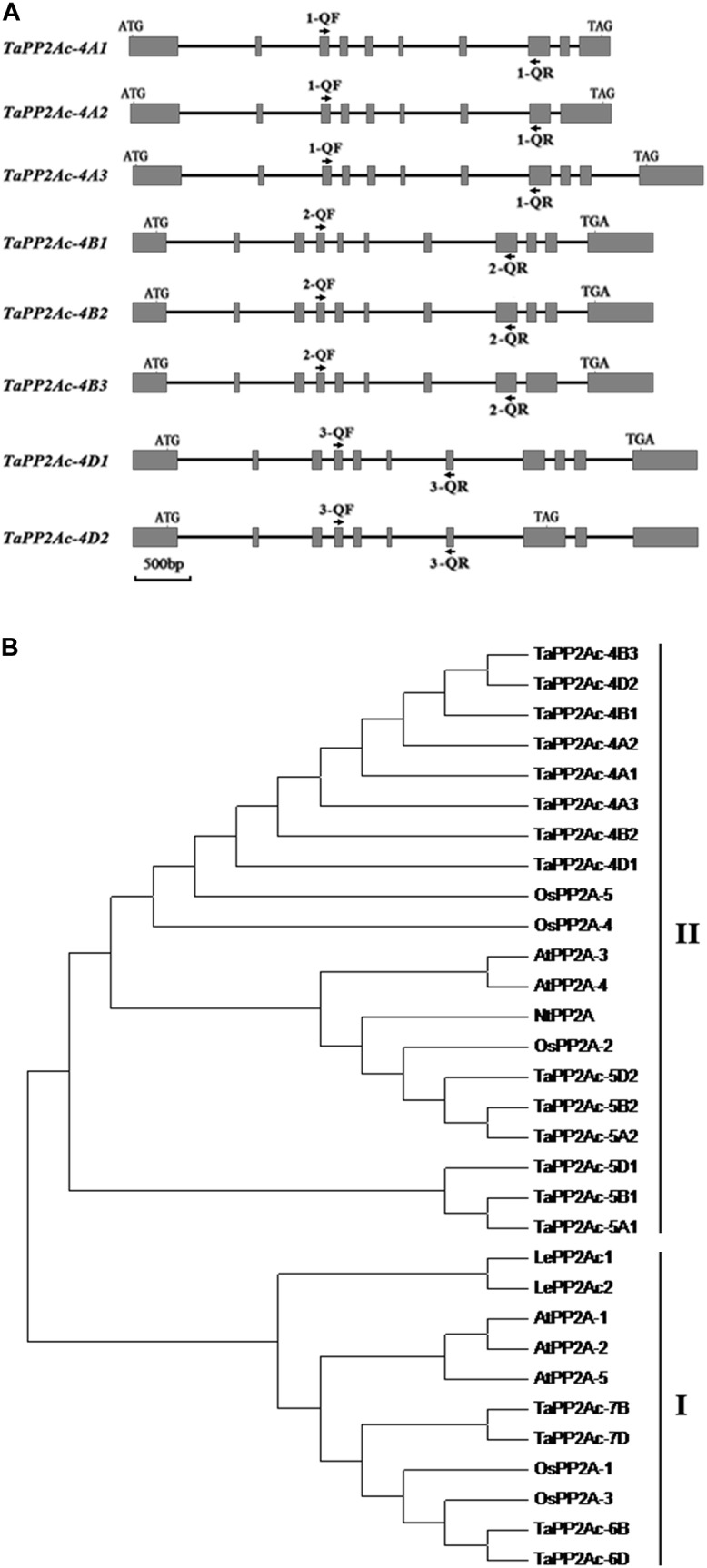
Gene structure and phylogenetic tree analysis of TaPP2Ac. **(A)** The diagram of gene structure of *TaPP2Ac*. Boxes and lines represent exons and introns, respectively. The black arrows indicate the primer sites for qRT-PCR to check the expression of *TaPP2Ac*. 1-QF stands for TaPP2Ac-4A-QF, 1-QR stands for TaPP2Ac-4A-QR, 2-QF stands for TaPP2Ac-4B-QF, 2-QR stands for TaPP2Ac-4B-QR, 3-QF stands for TaPP2Ac-4D-QF, and 3-QR stands for TaPP2Ac-4D-QR. The scale bar indicates gene length. **(B)** Neighbor-joining tree of protein phosphatase 2A catalytic subunit members from *Arabidopsis thaliana* (*At*), *Lycopersicon esculentum* (Le), *Nicotiana tabacum* (Nt), *Oryza sativa* (*Os*), and *Triticum aestivum* (*Ta*), generated by the MEGA6 software. The GenBank or Ensembl Plants accession numbers are as follows: OsPP2A-2 (A2XN40.2), OsPP2A-4 (A3C4N5.2), OsPP2A-5 (AAF86353.1), NtPP2A (Q9XGH7.1), AtPP2A-3 (NP_567066), and AtPP2A-4 (NP_973672), TaPP2Ac-5A1 (TRIAE_CS42_5AL_TGACv1_375154_AA1217070.1), TaPP2Ac-5A2 (TRIAE_CS42_5AL_TGACv1_376884_AA1242010.1), TaPP2Ac-5B1 (TRIAE_CS42_5BL_TGACv1_407550_AA1357850.1), TaPP2Ac-5B2 (TRIAE_CS42_5BL_TGACv1_405934_AA1337880.1), TaPP2Ac-5D1 (TRIAE_CS42_5DL_TGACv1_434113_AA1429600.1), TaPP2Ac-5D2 (TRIAE_CS42_5DL_TGACv1_433344_AA1410230.1), TaPP2Ac-6B (TRIAE_CS42_6BS_TGACv1_513814_AA1650260.1), TaPP2Ac-6D (TRIAE_CS42_6DS_TGACv1_544386_AA1748060.1), TaPP2Ac-7B (TRIAE_CS42_7BL_TGACv1_579107_AA1904240.1), TaPP2Ac-7D (TRIAE_CS42_7DL_TGACv1_605476_AA2006730.1).

Given that PP2Ac plays important roles in disease resistance in *Nicotiana benthamiana* (He et al., 2004), we were interested in elucidating the defense roles of PP2Ac in common wheat. Firstly, we cloned the homologous cDNA sequence of the *TaPP2Ac-4D* from the wheat lines/cultivars CI12633, Yangmai 16, and Wenmai 6. The cDNA sequence of *TaPP2Ac-4D* from the resistance wheat line CI12633 has been deposited in GenBank (accession number MG461318) with 987-bp length. Nucleic acid sequence analysis showed that no polymorphism exists between the cloned cDNA sequences from these three lines/cultivars. Common wheat is a hexaploid species with A, B, and D subgenomes. To explore the copy number and the chromosomal location of the *TaPP2Ac* gene, the gene sequence was aligned with the wheat cultivar Chinese spring (CS) genome sequence released in Ensembl Plants (see text footnote 1). This *TaPP2Ac* cDNA sequence hit three homologous genes that were located on chromosomes 4AS, 4BL, and 4DL, respectively. They were designated as *TaPP2Ac-4A*, *TaPP2Ac-4B*, and *TaPP2Ac-4D. TaPP2Ac-4A* and *TaPP2Ac-4B* had three transcripts, whereas *TaPP2Ac-4D* had two transcripts (Figure 1A). All copies of all *TaPP2Ac* genes possessed 9 to 11 exons based on annotation (Figure 1A). The major differences among the *TaPP2Ac* homologs were present in the 3′ end sequences (Figure 1A).

**FIGURE 2:**
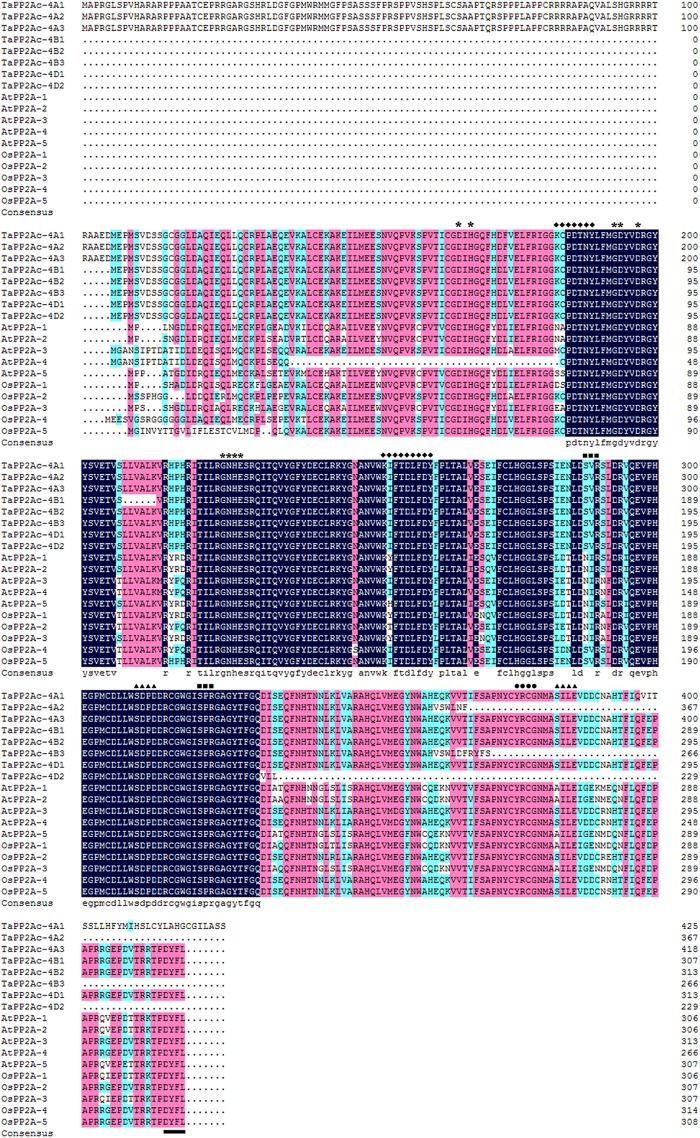
Multiple amino acid sequence alignment of TaPP2Ac with AtPP2A and OsPP2A. The software DANMAN was used to perform the sequence alignment. The serine/threonine specific protein phosphatase signature is marked by asterisks, the tyrosine kinase phosphorylation site is marked by diamonds, the protein kinase C phosphorylation site is marked by squares, the casein kinase II phosphorylation site is marked by triangles, the okadaic acid binding site is marked by spots, and the PP2A activity regulating site is marked by underline.

Alignments indicated that the amino acid sequences of different copies of TaPP2Ac shared 54.10 to 100.00% identities (Supplementary Table S2). Sequence analysis showed that the predicted ORFs of *TaPP2Ac-4A*, *TaPP2Ac-4B*, and *TaPP2Ac-4D* encoded proteins that are composed of 426, 306, and 313 amino acids, respectively. A phylogenetic analysis of TaPP2Ac-4A, TaPP2Ac-4B, and TaPP2Ac-4D with other PP2A proteins from wheat, *Arabidopsis*, rice, tobacco, and tomato revealed that TaPP2Ac-4A, TaPP2Ac-4B, and TaPP2Ac-4D clustered with OsPP2A-2, OsPP2A-4, OsPP2A-5, NtPP2A, AtPP2A-3, AtPP2A-4, TaPP2Ac-5A1, TaPP2Ac-5A2, TaPP2Ac-5B1, TaPP2Ac-5B2, TaPP2Ac-5D1, and TaPP2Ac-5D2, all of which are members of subfamily II according to the previous classification of PP2Ac proteins (Figure 1B; He et al., 2004). Multiple amino acid sequence alignment of TaPP2Ac copies with *Arabidopsis* and rice PP2Ac proteins showed that all the predicted TaPP2Ac proteins possessed the structure features of PP2Ac protein, including a Ser/Thr specific protein phosphatase signature, a tyrosine kinase phosphorylation site, a protein kinase C phosphorylation site, a casein kinase II phosphorylation site, an okadaic acid binding site, and a PP2A activity (Yu et al., 2005; País et al., 2010; Figure 2). These results suggest that TaPP2Ac-4A, TaPP2Ac-4B, and TaPP2Ac-4D are members of the PP2Ac protein in common wheat.

### *TaPP2Ac-4B* and *TaPP2Ac-4D* in Wheat Are Responsive to *R. cerealis* Infection

**FIGURE 3:**
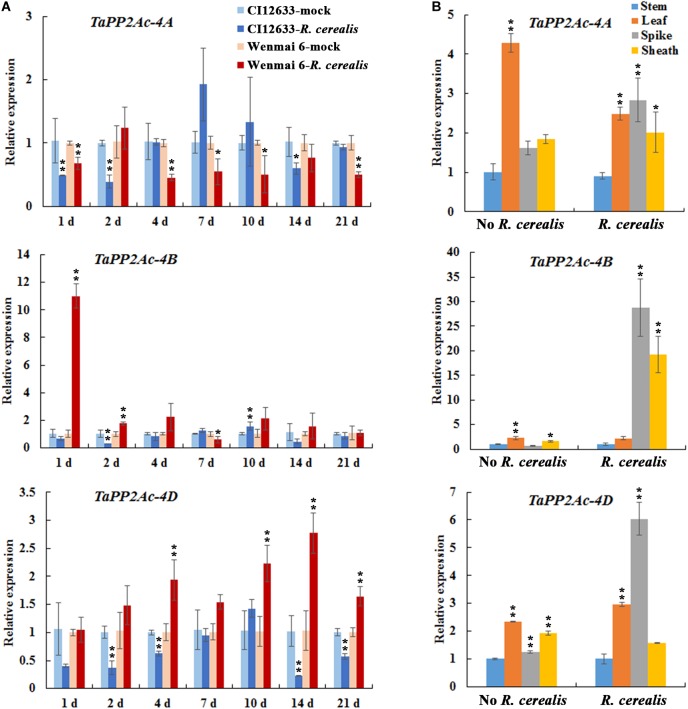
Transcription of *TaPP2Ac-4A*, *TaPP2Ac-4B*, and *TaPP2Ac-4D* in *Rhizoctonia cerealis*-inoculated wheat (*Triticum aestivum*). **(A)** Transcription of *TaPP2Ac-4A*, *TaPP2Ac-4B*, and *TaPP2Ac-4D* in the *R. cerealis*-resistant wheat line CI12633 and susceptible wheat cultivar Wenmai 6 at 1, 2, 4, 7, 10, and 21 days post inoculation (dpi) with *R. cerealis*. The expression level of *TaPP2Ac* in wheat plants treated by toothpick without *R. cerealis* mycelia (mock-inoculation) at each time point is set to 1. Statistically significant differences between *R. cerealis* treatment and mock at the same time point are derived from the results of three independent replications (*t*-test: ^∗∗^*P* < 0.01). **(B)** Transcription of *TaPP2Ac-4A*, *TaPP2Ac-4B*, and *TaPP2Ac-4D* in stems, leaves, spikes, and sheaths of wheat Yangmai 16 plants with inoculation by *R. cerealis* or mock treatment by sterile toothpicks without pathogen for 3 days. The transcriptional level of *TaPP2Ac* in stems with mock treatment was set to 1. Statistically significant differences are derived from the results of three independent replications (Dunnett’s test: ^∗^*P* < 0.05 and ^∗∗^*P* < 0.01). *TaActin* was used as the internal control.

To test the expression profiles of *TaPP2Ac*, we investigated the expression of *TaPP2Ac-4A*, *TaPP2Ac-4B*, and *TaPP2Ac-4D* in wheat after *R. cerealis* infection using qRT-PCR. As shown in Figure 3A, *TaPP2Ac-4A* transcriptional levels in susceptible wheat line Wenmai 6 were significantly reduced at 1–21 dpi except for 2 and 14 dpi; whereas, in resistant wheat line CI12633, the transcriptional levels of *TaPP2Ac-4A* were significantly reduced at 1 and 2 dpi. For *TaPP2Ac-4B* and *TaPP2Ac-4D*, in susceptible wheat line Wenmai 6, the transcriptional levels were upregulated as early as 1 dpi, with two peaks at 1 dpi (*TaPP2Ac-4B*) and 14 dpi (*TaPP2Ac-4D*) and then declined, although the levels were always higher than that of the mock-treated plants. However, in the resistant wheat line CI12633, the transcriptional levels of *TaPP2Ac-4B* were only elevated at 10 dpi and were relatively weaker; the transcriptional levels of *TaPP2Ac-4D* were downregulated at 1–21 dpi except for 10 dpi (Figure 3A). These data suggested that the expression of *TaPP2Ac-4B* and *TaPP2Ac-4D* rather than *TaPP2Ac-4A* was induced by *R. cerealis* infection in the susceptible wheat line Wenmai 6. Based on these observations, *TaPP2Ac-4B* and *TaPP2Ac-4D* were selected for further VIGS analysis during the response of wheat to *R. cerealis* infection.

We subsequently analyzed the expression pattern of *TaPP2Ac-4A*, *TaPP2Ac-4B*, and *TaPP2Ac-4D* in different tissues including stem, leaf, spike, and sheath of the wheat cultivar Yangmai 16. As shown in Figure 3B, without *R. cerealis* infection, *TaPP2Ac-4A*, *TaPP2Ac-4B*, and *TaPP2Ac-4D* were expressed in the highest levels in the leaves; whereas, at 3 dpi with *R. cerealis*, the transcriptional levels in spikes and/or leaves were more abundant than those in stems and sheaths, which were the main disease-occurring sites.

### *TaPP2Ac-4A*, *TaPP2Ac-4B*, and *TaPP2Ac-4D* Are Differentially Induced by H_2_O_2_ Treatment

**FIGURE 4:**
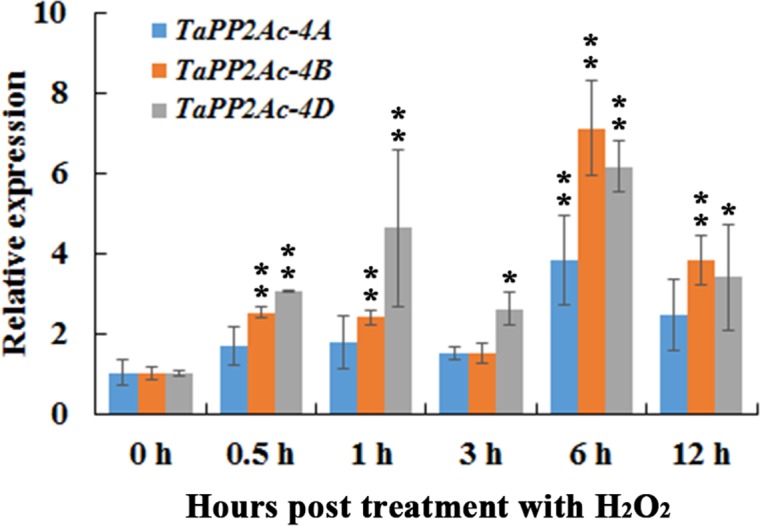
The transcription of *TaPP2Ac-4A*, *TaPP2Ac-4B*, and *TaPP2Ac-4D* in wheat response to H_2_O_2_ treatment. Deviation bars indicate SD among three independent replicates. The data were normalized to the wheat *TaActin* gene. The transcription level of *TaPP2Ac* in the control wheat plants (0 h) was set to 1. Asterisks indicate significant difference from 0 h after inoculation using Dunnett’s test (^∗^*P* < 0.05 and ^∗∗^*P* < 0.01).

In order to evaluate the possible linkages between immune signaling and H_2_O_2_, we investigated the transcription profiles of *TaPP2Ac-4A*, *TaPP2Ac-4B*, and *TaPP2Ac-4D* post treatment of H_2_O_2_ for 0.5, 1, 3, 6, and 12 h. As shown in Figure 4, when seedlings were treated with H_2_O_2_, the transcriptional levels of *TaPP2Ac-4B* and *TaPP2Ac-4D* were significantly elevated from 0.5 to 12 hpt, with an induction peak at 6 hpt (approximate 7.11-fold for *TaPP2Ac-4B* and 6.15-fold for *TaPP2Ac-4D*). The *TaPP2Ac-4A* transcriptional level was only upregulated at 6 hpt in response to H_2_O_2_ treatment (Figure 4).

### Silencing of *TaPP2Ac-4D* and *TaPP2Ac-4B* Increases Wheat Resistance to *R. cerealis*

**FIGURE 5:**
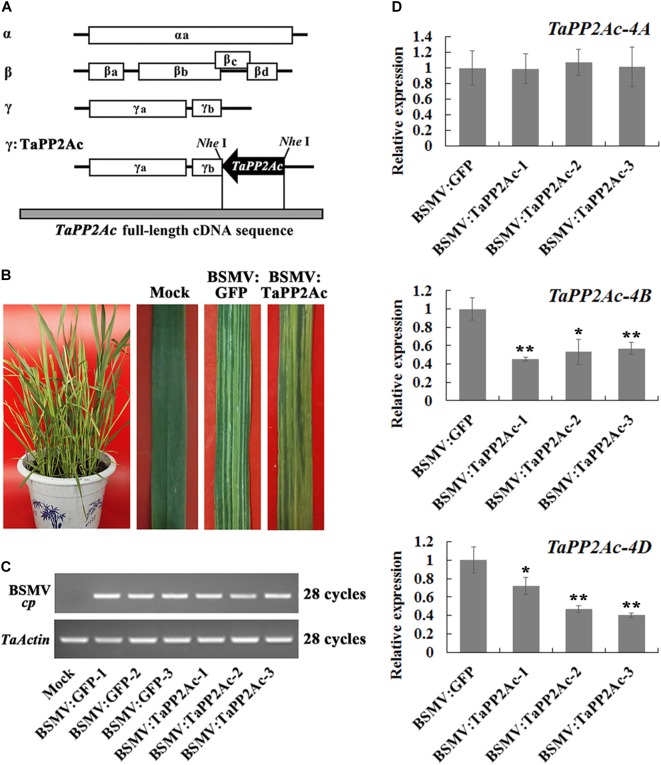
Silencing of *TaPP2Ac* by barley stripe mosaic virus (BSMV)-induced gene silencing in sharp eyespot moderately susceptible wheat Yangmai 16. **(A)** Scheme of genomic RNAs of BSMV construct and the construct of the recombinant virus expressing the wheat gene *TaPP2Ac*, BSMV:TaPP2Ac. The orientation of the *TaPP2Ac* insert is indicated by dark boxes. **(B)** Mild chlorotic mosaic symptoms observed on leaves at 7 days post inoculated (dpi) with BSMV:GFP or BSMV:TaPP2Ac. **(C)** RT-PCR analysis of the transcription levels of BSMV coat protein (*cp*) gene in the wheat plants infected by BSMV:GFP or BSMV:TaPP2Ac. **(D)** The relative transcript level of *TaPP2Ac-4A*, *TaPP2Ac-4B*, and *TaPP2Ac-4D* in BSMV:TaPP2Ac-infected wheat Yangmai 16 plants is relative to that seen in BSMV:GFP-infected (control) plants. BSMV:TaPP2Ac-1∼3 indicate mean value from three independent experiments. The transcript levels of three *TaPP2Ac* genes in BSMV:GFP-infected plants from three independent experiments were set to 1. At least 20 plants were infected by BSMV:GFP and BSMV:TaPP2Ac for one repeat. Significant differences were analyzed using Dunnett’s test (^∗^*P* < 0.05 and ^∗∗^*P* < 0.01). Deviation bars indicate SD.

To investigate whether TaPP2Ac plays a negative role in wheat defense response to *R. cerealis* infection, *TaPP2Ac* transcript was knocked down in the partial resistant wheat line CI12633 or in the moderately susceptible wheat line Yangmai 16 using BSMV-VIGS technique. Firstly, the VIGS construct used (Figure 5A) here was predicted by the Si-Fi software (see text footnote 3) to show more efficient siRNA hits on *TaPP2Ac-4B* and *TaPP2Ac-4D* compared with those on *TaPP2Ac-4A* (Supplementary Table S3), which suggested the efficient silencing of *TaPP2Ac-4B* and *TaPP2Ac-4D*. No off-target was predicted for the VIGS construct in wheat as determined by the Si-Fi software (Supplementary Table S3). At 7 dpi with BSMV, chlorotic mosaic phenotype was observed on the fourth leaves in both BSMV:TaPP2Ac- and BSMV:GFP-inoculated wheat Yangmai 16 plants (Figure 5B), and the expression of BSMV *cp* gene was clearly detected (Figure 5C), which indicated that these recombinant BSMV successfully infected wheat Yangmai 16 plants. To confirm silencing efficiency, the transcript levels of three copies of *TaPP2Ac* in stems of BSMV-infected wheat Yangmai 16 plants were evaluated by qRT-PCR. As shown in Figure 5D, the transcriptional levels of both *TaPP2Ac-4B* and *TaPP2Ac-4D* were markedly reduced in BSMV:TaPP2Ac-infected Yangmai 16 plants compared with the control plants inoculated with BSMV:GFP, whereas the transcriptional level of *TaPP2Ac-4A* was not downregulated. This was probably because the *TaPP2Ac* fragment sequence used for VIGS shared high identities with *TaPP2Ac-4D* (100%) and *TaPP2Ac-4B* (98.05%) but low identities with *TaPP2Ac-4A1* (71.81%) and *TaPP2Ac-4A2* (62.84%) (Supplementary Table S4).

**FIGURE 6:**
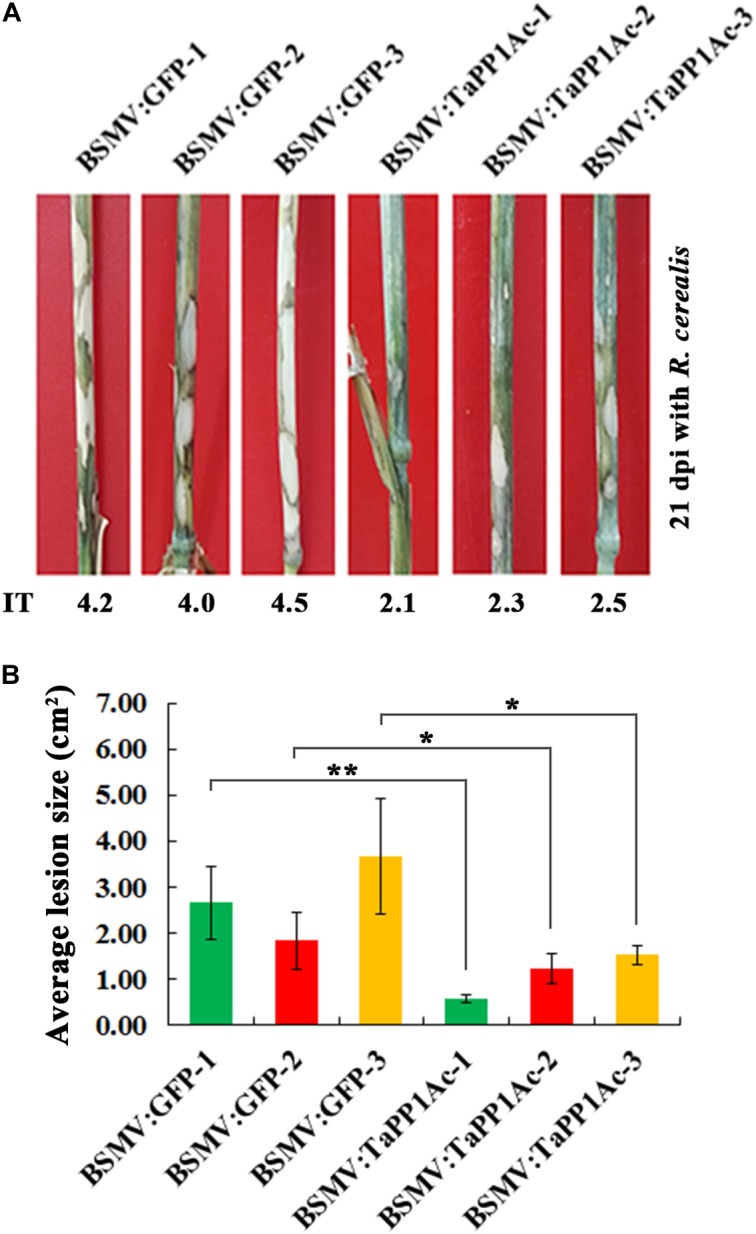
Silencing of *TaPP2Ac-4D* and *TaPP2Ac-4B* enhanced resistance of moderately susceptible wheat Yangmai 16 to *Rhizoctonia cerealis*. **(A)** Sharp eyespot symptoms of the control and *TaPP2Ac*-silenced Yangmai 16 plants at 21 dpi with *R. cerealis*. **(B)** Disease lesion size in *TaPP2Ac*-silenced and control Yangmai 16 plants at 21 dpi with *R. cerealis*. BSMV:GFP-1∼3 and BSMV:TaPP2Ac-1∼3 indicate mean value of each group from three independent replications. At least 20 plants were infected separately by BSMV:GFP and BSMV:TaPP2Ac for one repeat. Significant difference of BSMV:TaPP2Ac-infected wheat plants relative to the corresponding mean of control for each experiment was analyzed using Student *t*-test (^∗^*P* < 0.05 and ^∗∗^*P* < 0.01). Deviation bars indicate SD.

Subsequently, to evaluate the defense role of *TaPP2Ac*, these BSMV-infected Yangmai 16 wheat plants were further inoculated with the *R. cerealis* isolate WK207. After 21 days with *R. cerealis* inoculation, symptom of sharp eyespot disease was present on the stems of BSMV:GFP-infected control Yangmai 16 plants but to a lesser extent in BSMV:TaPP2Ac-infected Yangmai 16 plants (Figure 6A). The average ITs of BSMV:TaPP2Ac-infected Yangmai 16 plants ranged from 2.1 to 2.5, which were lower than that of BSMV:GFP-treated Yangmai 16 plants (ranged from 4.0 to 4.5). The disease lesion areas of BSMV:TaPP2Ac-infected Yangmai 16 plants were 0.56–1.53 cm^2^, whereas the disease lesion area on the stems of BSMV:GFP-infected Yangmai 16 plants were 1.83–3.67 cm^2^ (Figure 6B). Downregulation of *TaPP2Ac* in CI12633 wheat plants showed similar results (Supplementary Figure S1). The ITs of BSMV:TaPP2Ac-infected CI12633 plants ranged from 1.0 to 2.5, which were lower than those of BSMV:GFP-treated CI12633 plants (ranged from 2.5 to 4.0; Supplementary Figure S1). These results indicated that silencing of *TaPP2Ac-4B* and *TaPP2Ac-4D* in wheat increased host resistance to *R. cerealis* and suggested that TaPP2Ac-4B and TaPP2Ac-4D negatively regulated wheat resistance response to *R. cerealis* infection.

### *TaPP2Ac* Silencing Upregulates the Expression of *PR2* and ROS-Scavenging Enzyme Genes

**FIGURE 7:**
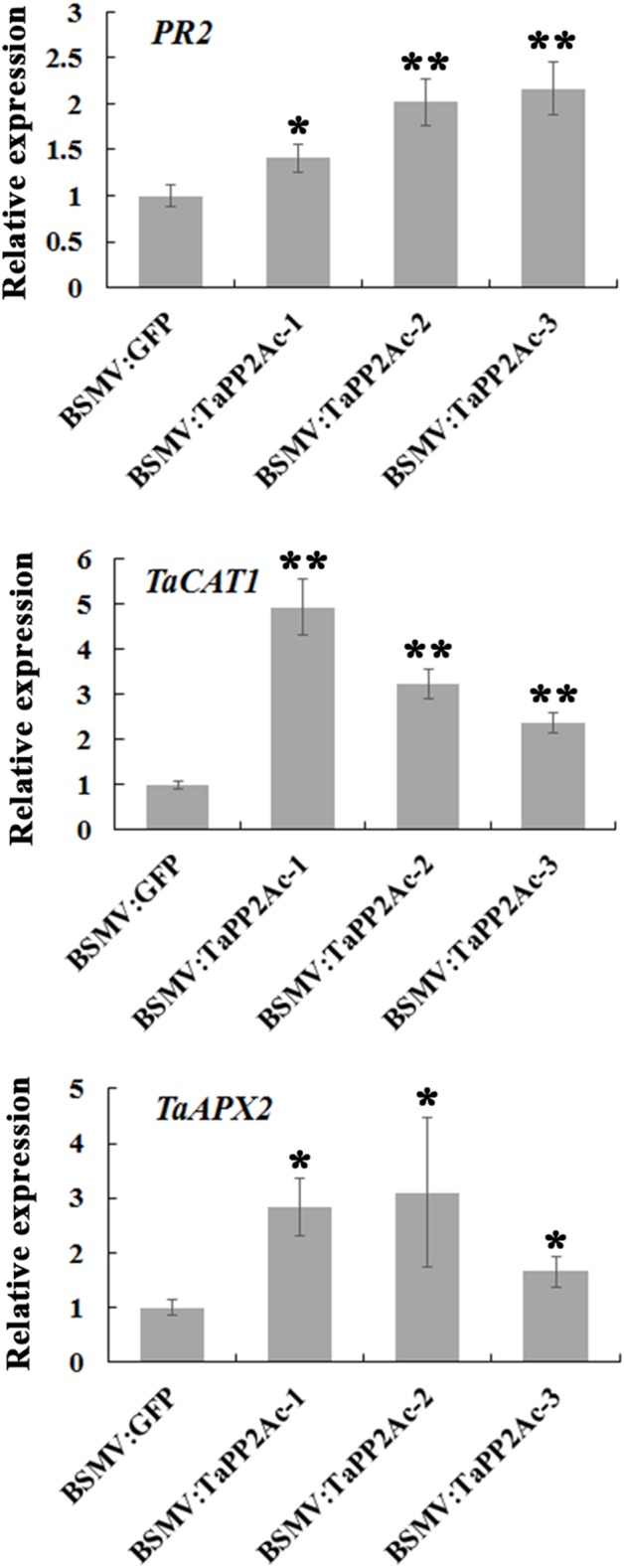
Transcriptional analysis of pathogenesis related gene *PR2* and ROS-scavenging related genes *TaCAT1* and *TaAPX2* in wheat Yangmai 16. The reported transcript levels of the tested gene in the BSMV:TaPP2Ac-infected wheat plants are relative to those seen in the BSMV:GFP-infected (control) plants. BSMV:TaPP2Ac-1∼3 indicate mean value from three independent experiments. The transcript levels of target genes in BSMV:GFP-infected plants from three independent experiments were set to 1. At least 20 plants were infected separately by BSMV:GFP and BSMV:TaPP2Ac for one repeat. Significant differences were analyzed using Dunnett’s test (^∗^*P* < 0.05 and ^∗∗^*P* < 0.01). Bars indicate standard deviation of the mean.

To examine whether TaPP2Ac regulates certain defense-associated genes in wheat response to *R. cerealis*, we analyzed the expression levels of several *PR* genes including *PR1*, *PR2*, *PR4*, *PR5*, *PR10*, and *PR17* in *TaPP2Ac*-silenced (BSMV:TaPP2Ac-infected) and control (BSMV:GFP-infected) plants (Figure 7; Supplementary Figure S2). As shown in Figure 7, *PR2* transcriptional level was higher in *TaPP2Ac*-silenced plants than that in control plants, suggesting that silencing of *TaPP2Ac* upregulated *PR2* transcription. To determine whether *TaPP2Ac* modulates H_2_O_2_ elimination, we examined the transcriptional levels of wheat genes encoding ROS-scavenging enzymes (CAT1 and APX2) in *TaPP2Ac*-silenced and control wheat plants. As a result, compared with control wheat plants, the transcriptional levels of *CAT1* and *APX2* were higher in *TaPP2Ac*-silenced plants than in the control plants (Figure 7). The results suggested that *TaPP2Ac* negatively regulated the transcriptional levels of *CAT1* and *APX2* genes.

## Discussion

In the current study, we proved that *TaPP2Ac-4B* and *TaPP2Ac-4D* act as negative regulators in the wheat defense response to *R. cerealis* infection. In plants, multiple genes encoding PP2A subunits were reported (Ariño et al., 1993; Kerk et al., 2002; Yu et al., 2005; País et al., 2009). For instance, in *Arabidopsis*, five genes encoding PP2Ac were reported (Ariño et al., 1993; Kerk et al., 2002). Since common wheat is a hexaploid species and composed of A, B, and D subgenomes, wheat genes theoretically have three homoeologous members, one per genome. For *TaPP2Ac*, we also found three homoeologous members located on chromosomes 4AS, 4BL, and 4DL. Comparison of the nucleotide sequences among *TaPP2Ac* homologs revealed some differences that mainly exist in the 3′ end sequences. These differences were predicted to lead to alternative splicing and affect the amino acid sequences of the resulting proteins. As supporting evidence for the alternative splicing, in common wheat, *TaPP2Ac-4A* and *TaPP2Ac-4B* each had three transcripts (*TaPP2Ac-4A1*, *TaPP2Ac-4A2*, and *TaPP2Ac-4A3* for *TaPP2Ac-4A*, and *TaPP2Ac-4B1*, *TaPP2Ac-4B2*, and *TaPP2Ac-4B3* for *TaPP2Ac-4B*), whereas *TaPP2Ac-4D* had only two transcripts (*TaPP2Ac-4D1* and *TaPP2Ac-4D2*).

A previous study found that in plants, PP2Ac proteins can be grouped into two subfamilies (He et al., 2004). Here, sequence and phylogenetic analyses indicate that TaPP2Ac-4A, TaPP2Ac-4B, and TaPP2Ac-4D proteins are typical members of PP2Ac subfamily II. Early studies have reported that plant PP2Ac proteins were involved in resistance responses to abiotic stresses (Yu et al., 2003, 2005; País et al., 2009; Liu et al., 2014; Hu et al., 2017) and bacterial pathogen infection (He et al., 2004). For instance, in *Arabidopsis*, *pp2a-c4* and *pp2a-a1* knock-out mutants exhibit enhanced resistance to the virulent pathogen *P. syringae* pv. *tomato* DC3000 (Segonzac et al., 2014). In this study, employing the BSMV-VIGS technique, the transcriptional levels of *TaPP2Ac-4B* and *TaPP2Ac-4D* were downregulated in wheat, whereas *TaPP2Ac-4A* transcription was not decreased. This is probably due to the low identities (62.84–71.81%) between the sequences of *TaPP2Ac-4A* and the fragmental sequence used in the construction of the VIGS vector. More importantly, knockdown of *TaPP2Ac-4B* and *TaPP2Ac-4D* enhanced wheat resistance to *R. cerealis* infection, indicating that TaPP2Ac negatively regulated the wheat defense response to *R. cerealis* infection. To the best of our knowledge, studies on the role of plant PP2Ac proteins in defense responses against necrotrophic pathogens have not been reported. Thus, this study is probably the first report that reveals the negative effect of a plant PP2Ac protein on defense response to necrotrophic fungal pathogens and broadens the understanding of biological functions of PP2Ac proteins in plant species.

By comparing this current report with previous functional studies on PP2Ac proteins, we can find a functional diversity between PP2Ac subfamily I and II in plants (Yu et al., 2003, 2005; He et al., 2004; País et al., 2009; Ballesteros et al., 2013; Liu et al., 2014; Hu et al., 2017). In brief, members of the PP2A subfamily I were usually involved in brassinosteroid and ABA signalings, as well as plant responses to salt and hemibiotropic pathogen stresses (He et al., 2004; Pernas et al., 2007; País et al., 2009; Tang et al., 2011; Hu et al., 2017), whereas members of the PP2A subfamily II likely play important roles in root development (Ballesteros et al., 2013) and in defense responses against necrotrophic fungal pathogens. The different functions may be partially due to the sequence differences. Even though the key residues and domains, such as the okadaic acid binding and PP2A activity regulating sites, are conserved in members of the two subfamilies, an important residue Asp284 that makes four intermolecular hydrogen bonds with the PP2A subunit A (Xing et al., 2006) shows divergence between the two subfamilies. The Asp284 is well conserved in PP2A subfamily II members (such as TaPP2Ac, AtPP2Ac-3, and AtPP2Ac-4) but is substituted with Glu284 in subfamily I members (such as AtPP2Ac-1, AtPP2Ac-2, and AtPP2Ac-5). The sequence variation probably leads to the binding specificity of the two PP2Ac subfamilies to PP2A subunit A. It will be interesting to further study how the sequence variation affects the functional diversity. Furthermore, in addition to PP2A subunit C encoded by five genes, the subunits A and B were also encoded by multiple genes (3 genes for subunit A, 17 genes for subunit B) in *Arabidopsi*s (Farkas et al., 2007), which could form at least 255 novel forms of PP2A (Hu et al., 2017). This characteristic may be another explanation for the functional diversity of PP2Ac proteins in plants.

Type 2A protein phosphatases, usually acting as regulators, participate in plant immune response (Durian et al., 2016). For example, in *Arabidopsis*, *pp2a-b*’γ mutant increased the abundance and phosphorylation levels of PR proteins including PR1, PR2, and PR5 (Trotta et al., 2011). In this study, TaPP2Ac was also found to have a negative effect on the expression of *PR2*. Silencing of *TaPP2Ac* upregulated the expression of *PR2*. This result was not in accordance with the report of He et al. (2004), according to which silencing of *NbNPP4-1* and *NbNPP4-2* activated the expression of *PR1a*, *PR1b*, and *PR5* genes in *N. benthamiana* (He et al., 2004). TaPP2Ac and NbNPP4 possibly act through different subsets of *PR* genes in plant defense responses to different life-type pathogens. Alternatively, another explanation is that TaPP2Ac belongs to subfamily II, whereas NbNPP4 is a member of subfamily I of the plant PP2Ac proteins (He et al., 2004). In plants, ROS is an important signaling molecule and is usually involved in signal transduction in numerous pathways (Rahikainen et al., 2016). During interaction between plants and pathogens, pathogen infection triggers oxidative bursts, which occur at the early stages of pathogen infection, subsequently induce hypersensitive cell death (Tenhaken et al., 1995; Garcia-Brugger et al., 2006). This is usually beneficial for plants as a mechanism to restrict the progression of invading pathogens, including biotrophs and hemibiotrophs (Torres et al., 2006; Lehmann et al., 2015). *R. cerealis* infection promoted the generation of ROS (Zhu et al., 2015). Catalase (CAT) is a major scavenging enzyme that quenches photorespiratory H_2_O_2_ in peroxisomes (Mhamdi et al., 2010). Enzymes that scavenge ROS and antioxidants play important roles in plant defense responses to certain necrotrophic pathogens (Kuźniak and Skłodowska, 2004; Sharma et al., 2007). Homeostasis of ROS, maintained by ROS-scavenging enzymes (such as CAT and ascorbate peroxidase 2, APX2) and ROS-producing enzyme was found to be crucial for role of TaAGC1 in wheat resistance response to the necrotrophic pathogen *R. cerealis* (Zhu et al., 2015). Segonzac et al. (2014) showed that PP2A led to the flagellin-induced ROS burst into the apoplast. In this study, *TaPP2Ac-4D* was responsive to H_2_O_2_ stimulus, suggesting that *TaPP2Ac-4D* may be involved in ROS signaling. Furthermore, the transcriptional levels of *CAT1* and *APX2* were significantly elevated in the *TaPP2Ac*-silenced wheat plants than in the control wheat plants, indicating that TaPP2Ac negatively regulated the expression of *CAT1* and *APX2*. These results are in line with the finding that the transcript level of *APX2* encoding a H_2_O_2_ scavenging enzyme increased in *pp2a-b*’γζ double mutants (Konert et al., 2015b); the finding that PP2A-B1 is required to regulate the transcript abundance of genes encoding antioxidant enzymes including mitochondrial AOX1A and 1D and monodehydroascorbate reductase 2 (MDAR2) and chloroplastic copper/zinc SOD 2 (CSD2) (Trotta et al., 2011; Li et al., 2014; Konert et al., 2015a).

In summary, wheat PP2Ac negatively regulated the expression of *PR2*, *CAT*, and *APX2*, leading to repression of host defense response against a necrotrophic pathogen *R. cerealis*. Thus, silencing of *TaPP2Ac-4B* and *TaPP2Ac-4D* enhances wheat resistance to *R. cerealis* infection. This study uncovers a novel function of the plant *PP2Ac* gene in plant immune responses and identifies a potential genetic resource for improving wheat resistance to *R. cerealis* using gene editing system.

## Author Contributions

ZZ and XZ designed the experiment and wrote the manuscript. XZ, YW, ZS, and LL performed the experiments and analyzed the data.

## Conflict of Interest Statement

The authors declare that the research was conducted in the absence of any commercial or financial relationships that could be construed as a potential conflict of interest.

## Footnotes

1 http://plants.ensembl.org/Triticum_aestivum/Info/Index?db=core

2 http://smart.embl-heidelberg.de/

3 http://www.snowformatics.com/si-fi.html

## Supplementary Material

Figure S1 Silencing of *TaPP2Ac* transcription enhanced resistance of wheat line CI12633 to *Rhizoctonia cerealis*. **(A)** qRT-PCR analysis of the transcriptional level of *TaPP2Ac* in the wheat plants infected by BSMV:GFP (control) or BSMV:TaPP2Ac. **(B)** Sharp eyespot symptoms of the BSMV:GFP- and BSMV:TaPP2Ac-infected CI12633 plants at 21 dpi with *R. cerealis*. **(C)** Disease lesion size in BSMV:GFP- and BSMV:TaPP2Ac-infected CI12633 plants at 21 dpi with *R. cerealis*. BSMV:GFP-1∼3 and BSMV:TaPP2Ac-1∼3 indicate mean value of each group from three independent replications. At least 20 plants were infected separately by BSMV:GFP and BSMV:TaPP2Ac for one repeat. Significant difference of BSMV:TaPP2Ac-infected wheat plants relative to the corresponding mean of control for each experiment was analyzed using Student *t*-test (^∗^*P* < 0.05 and ^∗∗^*P* < 0.01).

Figure S2 Transcriptional analysis of pathogenesis related genes in wheat Yangmai 16. The reported transcript levels of the tested gene in the BSMV:TaPP2Ac-infected wheat plants are relative to those in the BSMV:GFP-infected (control) plants. BSMV:TaPP2Ac-1∼3 indicate mean value from three independent experiments. The transcript levels of target genes in BSMV:GFP-infected plants from three independent experiments were set to 1. At least 20 plants were infected separately by BSMV:GFP and BSMV:TaPP2Ac for one repeat. Significant differences were analyzed using Dunnett’s test (^∗^*P* < 0.05 and ^∗∗^*P* < 0.01). Bars indicate standard deviation of the mean.

Table S1 Primers used in this study.

Table S2 The identities between amino acid sequences of different copies of TaPP2Ac.

Table S3 Prediction of *TaPP2Ac* off-target transcripts in wheat.

Table S4 The identities between the sequences of *TaPP2Ac* fragment used for VIGS and *TaPP2Ac* different copies.
