# Supplementary figures and images for: Silencing of the Wheat Protein Phosphatase 2A Catalytic Subunit TaPP2Ac Enhances Host Resistance to the Necrotrophic Pathogen Rhizoctonia cerealis
Source: Front Plant Sci. 2018 Oct 31;9:1437. doi: 10.3389/fpls.2018.01437 (PMC6220131; doi:10.3389/fpls.2018.01437)

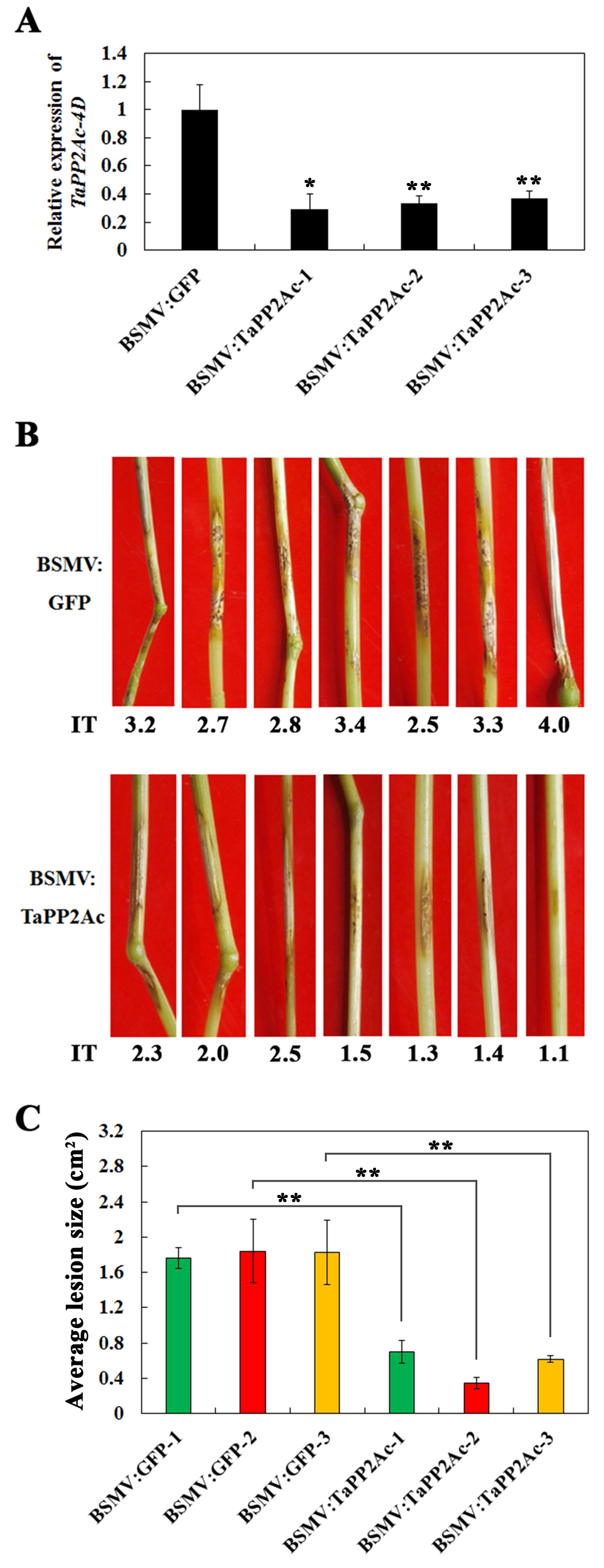

Supplement: Figure S1 — Silencing of TaPP2Ac transcription enhanced resistance of wheat line CI12633 to Rhizoctonia cerealis. (A) qRT-PCR analysis of the transcriptional level of TaPP2Ac in the wheat plants infected by BSMV:GFP (control) or BSMV:TaPP2Ac. (B) Sharp eyespot symptoms of the BSMV:GFP- and BSMV:TaPP2Ac-infected CI12633 plants at 21 dpi with R. cerealis. (C) Disease lesion size in BSMV:GFP- and BSMV:TaPP2Ac-infected CI12633 plants at 21 dpi with R. cerealis. BSMV:GFP-1∼3 and BSMV:TaPP2Ac-1∼3 indicate mean value of each group from three independent replications. At least 20 plants were infected separately by BSMV:GFP and BSMV:TaPP2Ac for one repeat. Significant difference of BSMV:TaPP2Ac-infected wheat plants relative to the corresponding mean of control for each experiment was analyzed using Student t-test (∗P < 0.05 and ∗∗P < 0.01). [file Image_1.TIF]

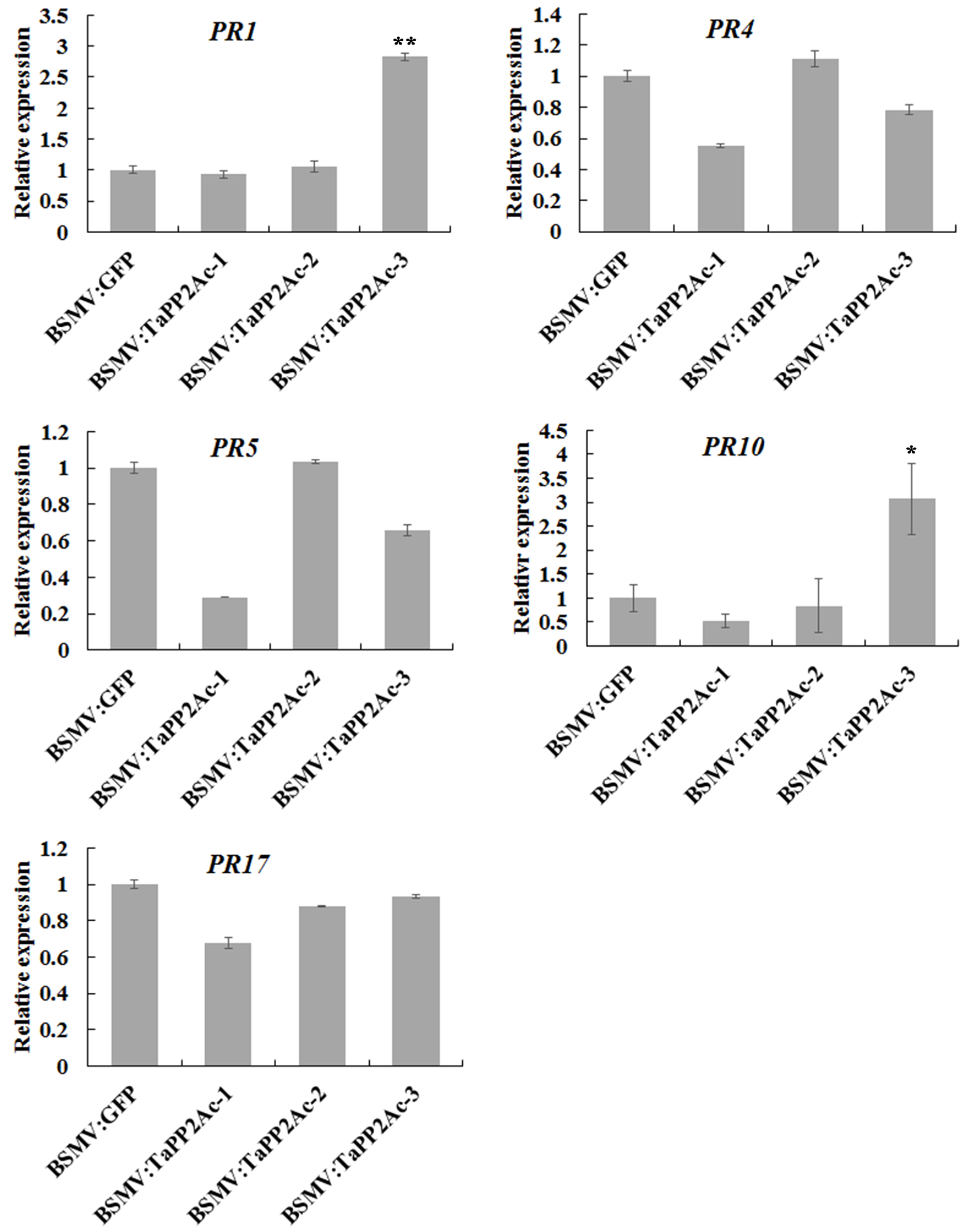

Supplement: Figure S2 — Transcriptional analysis of pathogenesis related genes in wheat Yangmai 16. The reported transcript levels of the tested gene in the BSMV:TaPP2Ac-infected wheat plants are relative to those in the BSMV:GFP-infected (control) plants. BSMV:TaPP2Ac-1∼3 indicate mean value from three independent experiments. The transcript levels of target genes in BSMV:GFP-infected plants from three independent experiments were set to 1. At least 20 plants were infected separately by BSMV:GFP and BSMV:TaPP2Ac for one repeat. Significant differences were analyzed using Dunnett’s test (∗P < 0.05 and ∗∗P < 0.01). Bars indicate standard deviation of the mean. [file Image_2.TIF]
