# Supplementary material for: Silencing of the Wheat Protein Phosphatase 2A Catalytic Subunit TaPP2Ac Enhances Host Resistance to the Necrotrophic Pathogen Rhizoctonia cerealis
Source: Front Plant Sci. 2018 Oct 31;9:1437. doi: 10.3389/fpls.2018.01437 (PMC6220131; doi:10.3389/fpls.2018.01437)
Supplement: Table S1 — Primers used in this study. [file Table_1.DOCX]

**Table S1** Primers used in this study

| Primer name | Accession number | Sequence of  gene-specific primer | Usage |
| --- | --- | --- | --- |
| TaPP2Ac-FL-F1 | EF101900.1 | 5′- GCGTGAGAGGACATGGAGCC -3′ | Amplification of full-length cDNA of *TaPP2Ac* |
| TaPP2Ac-FL-R1 |  | 5′- CACTGATCGGTGCGGACTAT -3′ |  |
| TaPP2Ac-FL-F2 |  | 5′- ATGGAGCCCATGAGCGTG -3′ |  |
| TaPP2Ac-FL-R2 |  | 5′- GCGGACTATCGCAATACAAGC -3′ |  |
| TaPP2Ac-γ-F | MG461318 | 5′-GACGCTAGCTCATCAGTTAGTTATGGAGGGA-3′ | Construction of vector γ- *TaPP2Ac* |
| TaPP2Ac-γ-R |  | 5′-TACGCTAGCGCGGACTATCGCAATACAAG-3′ |  |
| BSMV-CP-F | JF803284 | 5′-TGACTGCTAAGGGTGGAGGA-3′ | Detection of BSMV virus |
| BSMV-CP-R |  | 5′-CGGTTGAACATCACGAAGAGT-3′ |  |
| TaActin-F | BE425627 | 5′-CACTGGAATGGTCAAGGCTG-3′ | Internal control for qRT-PCR |
| TaActin-R |  | 5′-CTCCATGTCATCCCAGTTG-3′ |  |
| TaPP2Ac-4A-QF | TRIAE_CS42_4AS_TGACv1_  307022_AA1016250 | 5’- ATTCACGGACAATTCCATGATT-3’ | qRT-PCR for *TaPP2Ac-4A* transcript |
| TaPP2Ac-4A-QR |  | 5’- ATGGAGATAAACCACCATGCAA-3’ |  |
| TaPP2Ac-4B-QF | TRIAE_CS42_4BL_TGACv1_  320988_AA1053050 | 5’- TTATTTGTTTATGGGGGATTAC-3’ | qRT-PCR for *TaPP2Ac-4B* transcript |
| TaPP2Ac-4B-QR |  | 5’- ACCATAAAAGATCACACATAGGA-3’ |  |
| TaPP2Ac-4D-QF | MG461318 | 5’- TGTGGATCGTGGATACTACTCC -3’ | qRT-PCR for *TaPP2Ac-4D* transcript |
| TaPP2Ac-4D-QR |  | 5’- ATACATTTGCATTGCCGTACTTC-3’ |  |
| TaCAT1-QF | GU984379 | 5′-CAAGGGCTTCTTCGAGGTCAC-3′ | qRT-PCR for *TaCAT1* transcript |
| TaCAT1-QR |  | 5′- TGTAGAAGGTCCACTCCGGGTAG-3′ |  |
| TaAPX2-QF | AK332842 | 5′- GACGGTCAGCGACGAGTA-3′ | qRT-PCR for *TaAPX2* transcript |
| TaAPX2-QR |  | 5′- GTGGTCAGAGCCTTGGGT-3′ |  |
| PR1-QF | AJ007349 | 5′- CGTCTTCATCACCTGCAACTA-3′ | qRT-PCR for *PR1* transcript |
| PR1-QR |  | 5′- CAAACATAAACACACGCACGTA-3′ |  |
| PR2-QF | AF112965 | 5′- CCGCACAAGACACCTCAAGATA-3′ | qRT-PCR for *PR2* transcript |
| PR2-QR |  | 5′- CGATGCCCTTGGTTTGGTAGA-3′ |  |
| PR4-QF | AJ006099.1 | 5′-ACACCGTCTTCACCAAGATCGACA-3′ | qRT-PCR for *PR4* transcript |
| PR4-QR |  | 5′-AGCATGGATCAGTCTCAGTGCTCA-3′ |  |
| PR5-QF | AF442967.1 | 5′-ACAGCTACGCCAAGGACGAC-3′ | qRT-PCR for *PR5* transcript |
| PR5-QR |  | 5′-CGCGTCCTAATCTAAGGGCAG-3′ |  |
| PR10-QF | CA613496 | 5’- CGTGGAGGTAAACGATGAG-3’ | qRT-PCR for *PR10* transcript |
| PR10-QR |  | 5’- GCTAAGTGTCCGGGGTAAT-3’ |  |
| PR17-QF | TRIAE_CS42_6AS_TGACv1_  486422_AA1561090.1 | 5’- CGACGACTTCGCAGATTCTC-3’ | qRT-PCR for *PR17* transcript |
| PR17-QR |  | 5’- GTGTGGTATGCCATCTCACG-3’ |  |
