# Supplementary material for: Silencing of the Wheat Protein Phosphatase 2A Catalytic Subunit TaPP2Ac Enhances Host Resistance to the Necrotrophic Pathogen Rhizoctonia cerealis
Source: Front Plant Sci. 2018 Oct 31;9:1437. doi: 10.3389/fpls.2018.01437 (PMC6220131; doi:10.3389/fpls.2018.01437)
Supplement: Table S2 — The identities between amino acid sequences of different copies of TaPP2Ac. [file Table_2.DOCX]

**Table S2** Percentage of amino acid identity among copies of wheat TaPP2Ac.

| **Identity (%)** | **TaPP2Ac-4A1** | **TaPP2Ac-4A2** | **TaPP2Ac-4A3** | **TaPP2Ac-4B1** | **TaPP2Ac-4B2** | **TaPP2Ac-4B3** | **TaPP2Ac-4D1** | **TaPP2Ac-4D2** |
| --- | --- | --- | --- | --- | --- | --- | --- | --- |
| **TaPP2Ac-4A1** |  |  |  |  |  |  |  |  |
| **TaPP2Ac-4A2** | 85.25 |  |  |  |  |  |  |  |
| **TaPP2Ac-4A3** | 93.44 | 87.54 |  |  |  |  |  |  |
| **TaPP2Ac-4B1** | 63.28 | 61.62 | 74.07 |  |  |  |  |  |
| **TaPP2Ac-4B2** | 69.18 | 62.63 | 70.03 | 98.65 |  |  |  |  |
| **TaPP2Ac-4B3** | 61.64 | 70.79 | 63.30 | 82.96 | 84.30 |  |  |  |
| **TaPP2Ac-4D1** | 69.18 | 62.63 | 75.08 | 98.65 | 100 | 84.30 |  |  |
| **TaPP2Ac-4D2** | 54.10 | 63.50 | 55.56 | 72.65 | 73.99 | 85.49 | 73.99 |  |
