# Supplementary material for: Silencing of the Wheat Protein Phosphatase 2A Catalytic Subunit TaPP2Ac Enhances Host Resistance to the Necrotrophic Pathogen Rhizoctonia cerealis
Source: Front Plant Sci. 2018 Oct 31;9:1437. doi: 10.3389/fpls.2018.01437 (PMC6220131; doi:10.3389/fpls.2018.01437)
Supplement: Table S3 — Prediction of TaPP2Ac off-target transcripts in wheat. [file Table_3.DOCX]

**Table S3** Prediction of *TaPP2Ac* off-target transcripts in wheat^a^

| **Targets** | **Total siRNA hits^b^** | **Efficient siRNA hits^c^** |
| --- | --- | --- |
| TRIAE_CS42_4BL_TGACv1_320988_AA1053050.1 (*TaPP2Ac-4B1*) | 236 | 124 |
| TRIAE_CS42_4BL_TGACv1_320988_AA1053050.2 (*TaPP2Ac-4B2*) | 236 | 124 |
| TRIAE_CS42_4BL_TGACv1_320988_AA1053050.3 (*TaPP2Ac-4B3*) | 217 | 112 |
| TRIAE_CS42_4DL_TGACv1_342818_AA1122930.1 (*TaPP2Ac-4D1*) | 145 | 75 |
| TRIAE_CS42_4DL_TGACv1_342818_AA1122930.2 (*TaPP2Ac-4D2*) | 145 | 75 |
| TRIAE_CS42_4AS_TGACv1_307022_AA1016250.1 (*TaPP2Ac-4A1*) | 93 | 46 |
| TRIAE_CS42_4AS_TGACv1_307022_AA1016250.2 (*TaPP2Ac-4A2*) | 74 | 34 |
| TRIAE_CS42_4AS_TGACv1_307022_AA1016250.3 (*TaPP2Ac-4A3*) | 127 | 62 |
| TRIAE_CS42_5AL_TGACv1_375154_AA1217070.1 | 3 | 0 |
| TRIAE_CS42_5BL_TGACv1_407550_AA1357850.1 | 3 | 0 |
| TRIAE_CS42_5DL_TGACv1_434113_AA1429600.1 | 3 | 0 |

^a^ The off-target prediction was performed by using the Si-Fi software.

^b^ Number of 21-mer siRNA sequences with perfect match to the query sequence.

^c^ Number of 21-mer siRNA sequences with perfect match to the query sequence that fulfil additional criteria for efficient RNAi (see Si-Fi software, http://www.snowformatics.com/si-fi.html).
