# Supplementary material for: Silencing of the Wheat Protein Phosphatase 2A Catalytic Subunit TaPP2Ac Enhances Host Resistance to the Necrotrophic Pathogen Rhizoctonia cerealis
Source: Front Plant Sci. 2018 Oct 31;9:1437. doi: 10.3389/fpls.2018.01437 (PMC6220131; doi:10.3389/fpls.2018.01437)
Supplement: Table S4 — The identities between the sequences of TaPP2Ac fragment used for VIGS and TaPP2Ac different copies. [file Table_4.DOCX]

**Table S4** The identities between the sequences of *TaPP2Ac* fragment used for VIGS and *TaPP2Ac* copies

| ***TaPP2Ac*** | **Identity (%)** |
| --- | --- |
| *TaPP2Ac-4A1* | 71.81 |
| *TaPP2Ac-4A2* | 62.84 |
| *TaPP2Ac-4A3* | 97.27 |
| *TaPP2Ac-4B1* | 98.05 |
| *TaPP2Ac-4B2* | 98.05 |
| *TaPP2Ac-4B3* | 98.05 |
| *TaPP2Ac-4D1* | 100 |
| *TaPP2Ac-4D2* | 100 |
